# Fractal Hypothesis of the Pelagic Microbial Ecosystem—Can Simple Ecological Principles Lead to Self-Similar Complexity in the Pelagic Microbial Food Web?

**DOI:** 10.3389/fmicb.2015.01357

**Published:** 2015-12-01

**Authors:** Selina Våge, T. Frede Thingstad

**Affiliations:** Marine Microbial Ecology Group, Department of Biology, University of Bergen and Hjort Centre for Marine Ecosystem DynamicsBergen, Norway

**Keywords:** pelagic microbial food web, complexity at different scales, fractal-like organization, Sierpinski triangle, underlying ecological mechanisms, killing-the-winner

## Abstract

Trophic interactions are highly complex and modern sequencing techniques reveal enormous biodiversity across multiple scales in marine microbial communities. Within the chemically and physically relatively homogeneous pelagic environment, this calls for an explanation beyond spatial and temporal heterogeneity. Based on observations of simple parasite-host and predator-prey interactions occurring at different trophic levels and levels of phylogenetic resolution, we present a theoretical perspective on this enormous biodiversity, discussing in particular self-similar aspects of pelagic microbial food web organization. Fractal methods have been used to describe a variety of natural phenomena, with studies of habitat structures being an application in ecology. In contrast to mathematical fractals where pattern generating rules are readily known, however, identifying mechanisms that lead to natural fractals is not straight-forward. Here we put forward the hypothesis that trophic interactions between pelagic microbes may be organized in a fractal-like manner, with the emergent network resembling the structure of the Sierpinski triangle. We discuss a mechanism that could be underlying the formation of repeated patterns at different trophic levels and discuss how this may help understand characteristic biomass size-spectra that hint at scale-invariant properties of the pelagic environment. If the idea of simple underlying principles leading to a fractal-like organization of the pelagic food web could be formalized, this would extend an ecologists mindset on how biological complexity could be accounted for. It may furthermore benefit ecosystem modeling by facilitating adequate model resolution across multiple scales.

## Introduction

The pelagic is among the world's largest biomes and its microbes comprise the world's oldest living community. As major drivers of biogeochemical cycles, marine microbes are fundamental for climate relevant processes and they form the basis for marine harvestable resources. They furthermore play a key role in eutrophication events and act as bioremediators after oil spills. Understanding the functioning of this important community is thus subject to intensive research, and many questions related to regulating processes and impacts of environmental change remain open. Perhaps an even more fundamental biological question regards the enormous biodiversity that is generated and maintained in the pelagic microbial community. Sequencing techniques developed since the 1990s (Giovannoni et al., 1990) reveal an overwhelming and unprecedented amount of microbial diversity, and much of modern microbial ecology focuses on mapping this fascinating diversity (Rappé and Giovannoni, 2003; Martiny et al., 2006). It appears to us that the development of theory to account for this diversity has been somewhat lagging behind. More than ever before, the rapidly expanding knowledge of diversity demands explaining (Prosser et al., 2007).

Classic ecological theory including the niche (Grinnell, 1904) and competitive exclusion principles (Gause, 1934) state that a resource will be monopolized by the most competitive consumer. The consequence of this would be that in a relatively homogenous environment, a few, efficient and ecologically distinct species would dominate. On this basis, the “paradox of the plankton” (Hutchinson, 1961) has been described as an apparent conflict between many different phytoplankton species coexisting and a seemingly homogenous pelagic environment. We later learned that the pelagic environment is more heterogeneous than previously assumed, with particle aggregates structuring it on the small scale (Silver et al., 1978). In addition to spatial and temporal heterogeneity, an important mechanism allowing for coexistence of imbalanced competitors is selective top-down control of the competitively superior group, which is central to our proposed hypothesis of a fractal-like organization of the pelagic food web. Selective top-down control lies at the heart of the well known principle of key-stone predation in macro-ecology (Paine, 1966; Leibhold, 1996) and has been formalized under the “Killing-the-Winner” (KtW) principle (Thingstad, 2000) in microbial ecology. KtW depends inherently on a trade-off between competitive and defensive abilities among the competing groups, as a super-organism combining superior competition with superior defense would otherwise have evolved monopolizing all available resources.

The aim of this paper is to broaden the theoretical perspective on the overwhelming microbial diversity in the pelagic ecosystem and discuss potential underlying mechanisms. Searching for unifying mechanisms in biology should be as warranted and common practice as in physics, where a tight coupling between experimental data and theory development fosters a deep understanding of physical mechanisms. The additional degree of complexity that biology imposes onto the physical world may explain why development of theory in biology has been somewhat lagging behind. Understanding evolution and its underlying mechanisms (Darwin, 1859) has been one of the most significant achievements in unifying biology. Recently, motivated by patterns at the levels of populations, communities and ecosystems that emerge from traits at the level of individuals (Marquet et al., 2005), efforts have also been intensified to formulate a theory unifying traditionally distinct biological disciplines such as physiology, ecology, biogeography and macro-evolution (e.g., Brown et al., 2004). Energy flow and nutrient cycling in ecosystems are influenced by such emergent patterns, which can often be depicted as power laws (Peters, 1983; Halley et al., 2004). Important examples of such patterns are size structures and biomass distributions. However, even though body size is recognized as a master trait linking physiological, ecological and evolutionary patterns (Woodward et al., 2005), the existence of universal principles from which a general theory of biology can be established remains debated (Scheiner and Willig, 2008).

We here consider the confined subject of biological complexity in the pelagic microbial ecosystem and discuss how relatively simple ecological principles may account for much of the network organization, evolutionary dynamics and resulting biodiversity in this system. Based on observations of the KtW mechanism acting repeatedly at different trophic levels and levels of phylogenetic resolution, we provide a perspective on how this may give rise to a complex, fractal-like organization across different scales within the pelagic food web. A brief introduction to fractal geometry and characteristics of fractals is given in Box 1. A consequence of a fractal-like organization of trophic interactions for modeling would be that adequate resolution of several trophic levels would be facilitated. Although the applicability of this novel approach remains to be seen, the KtW mechanism acting repeatedly across at least four orders of magnitude in cell size (Finkel et al., 2010) makes this a promising question to pursue. Scale-invariance apparent in pelagic biomass-size spectra, originally proposed by Sheldon et al. (1972), seems utterly supportive of a fractal-hypothesis of the pelagic microbial food web. Whereas power-law dispersal and fractal branching networks have been used successfully to describe spatial and temporal patterns in macro-ecology and vascular systems in organic structures, respectively (Sugihara and May, 1990; Pascual et al., 1995; Halley et al., 2004), understanding trophic networks by means of fractal geometry has to the best of our knowledge not been attempted before.

Box 1 **Fractal characterization based on self-similarity and fractal dimensions.**

Fractals are mathematical structures that have the same structural complexity and detail at any scale. In other words, fractals have self-similarities at any scale (i.e., they are scale-invariant), and the hierarchic structure of fractals may be constructed by iteratively applying simple rules ad infinitum (Mandelbrot and Blumen, 1989). A famous example of a geometrical fractal is the Sierpinsky triangle that has a missing central triangle at each level of resolution (**Figure 6B**). Fractals are characterized by their fractal dimension, which for geometric fractals can be calculated as

$$\begin{array}{l} D=\frac{log\left(N\right)}{log\left(1∕\epsilon \right)}, \end{array}$$

where *N* is the number of downscaled copies at a particular scale and ϵ is the scaling factor of the downscaled copies (Mandelbrot et al., 1985). Due to the self-similarity at all scales, fractal dimensions fall somewhere between the classical Euclidean dimensions of either a line (1 dimension), a plane (2 dimensions), or a volume (3 dimensions). Coast lines and organic branching structures such as trees and leaves are examples of natural fractals, which have an approximate self-similarity within a confined range of scales.

We note that a fractal approach to understanding trophic interactions in the pelagic microbial community appears particularly fruitful, since high evolutionary rates among microbes overlap ecological time scales and the physical environment has been relatively stable over long periods of time. This is more likely to promote a mature state in the microbial community, where an equilibrium in terms of diversification processes at different levels of phylogenetic resolution (including strains, species and PFTs) could be reached. In contrast, macro-faunal communities have complex and long life-histories including large-scale migration, which may prevent the system from reaching an equilibrium on the large scale. Also, reaching a mature state where the full potential of evolutionary diversification is reached within the longer time scales acting on macro-faunal communities may be prevented by catastrophic events that disrupt and set the system back to an immature state (Stanley, 1973).

### Food web complexities at different scales

The pelagic microbial food web is characterized by different levels of complexity at different scales. On the large scale, the biogeographical distribution of microorganisms is given by chemical and physical parameters (Follows et al., 2007), setting the stage for secondary production and pelagic fish distribution. Locally, different plankton functional types such as calcifiers and silicifiers coexist in a food web by filling particular biogeochemical niches (LeQuéré et al., 2005). Within a particular group, size selective grazing (Gonzalez et al., 1990; Hahn and Höfle, 1999; Ward et al., 2013) and strain-specific viral control (Thingstad and Lignell, 1997; Fuhrman, 1999; Rodriguez-Valera et al., 2009) may promote diversity and coexistence of different species and “clonal” strains.

**Figure 1:**
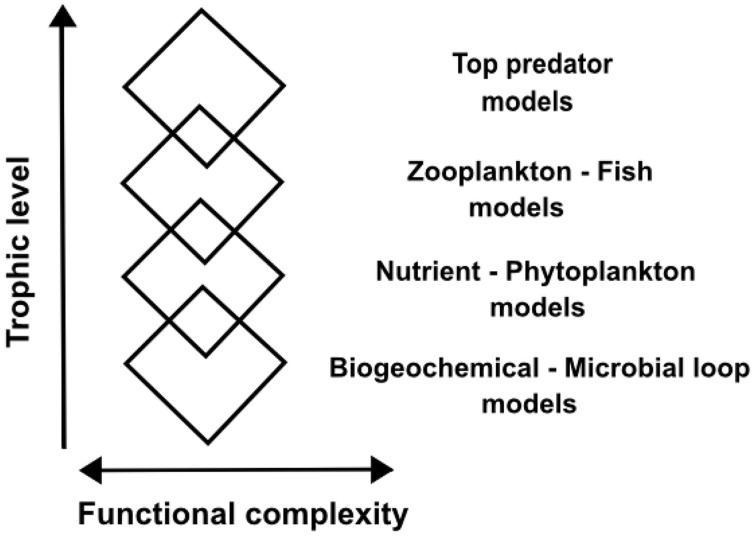
**Schematic of “rhomboidal modeling” of marine ecosystems**. The rhomboid width translates into functional complexity of a particular trophic level represented in a model, and overlapping rhomboids illustrate coupling of models of different trophic levels. Modified from de Young et al. (2004).

This structured complexity imposes a challenge for pelagic ecosystem modeling. One common way of tackling this complexity of pelagic ecosystems in ocean modeling is the “rhomboidal modeling” approach (de Young et al., 2004), where a particular trophic level is finely resolved in its functional complexity, while trophic levels below and above the level of interest are included with much reduced detail (Figure 1). This allows a focused study of processes on a particular trophic level but lacks a holistic approach to ecosystem understanding.

Another well-established approach is to reduce complexity to one functional group per trophic level, resulting in nutrient-phytoplankton-zooplankton-models (Steele and Henderson, 1992). Such models are a valuable tool in oceanography (Franks, 2002), but plankton dynamics are poorly resolved and important ecological processes such as temperature dependent bacterial remineralization (Rivkin and Legendre, 2001) and grazing rates (Buitenhuis et al., 2006) are ignored (LeQuéré, 2006; Mitra et al., 2007). Models resolving several different plankton functional types, including e.g., nitrogen-fixers, calcifiers, mixotrophs, protozoa, and mesozooplankton (Anderson, 2005; LeQuéré, 2006) resolve ecosystem dynamics more adequately but require parameter fitting, often with a lack of a clear understanding of mechanisms underlying the parameters (Fasham et al., 1990). Arguably, biologically most realistic are models where the environment selects favorable combinations of traits (Follows et al., 2007; Follows and Dutkiewicz, 2011; Ward et al., 2012) and where foraging responses emerge based on the criterion of evolutionary fitness at the level of individuals (Visser et al., 2012; Visser and Fiksen, 2013). Such models avoid the problem of ecologically unjustified parameter fitting, and they reflect the idea that patterns at the population and community level emerge from selected traits at the individual level (Stillman et al., 2015). An important aspect in such models are trade-offs associated with organism traits, whose mechanisms may be key for understanding how food web structures emerge (Litchman et al., 2015). Central for the KtW mechanism suggested here to lead to a fractal-like organization of the pelagic microbial food web is a trade-off between competitive and defensive abilities of pelagic microbes. Although a mechanistic understanding of such trade-offs is still very limited and experimental work is required to elaborate these trade-offs, it has for instance been shown in phage-host systems that cost of resistance against viral infections allows coexistence of susceptible and resistant hosts (Lenski, 1988; Buckling and Rainey, 2002; Lennon et al., 2007; Avrani et al., 2011).

## Fractal hypothesis for the pelagic microbial food web

According to the fractal hypothesis of the pelagic microbial food web put forward here, different trophic levels are controlled in similar manners within their respective characteristic temporal and spatial scales. An understanding of organization at one level would thus give a basic understanding of organization at all other levels. If this concept is valid and can be formalized, it would be an innovative way of considering phylogenetic diversity and food web complexity. Furthermore, “end-to-end” ecosystem modeling (Travers et al., 2007; Fulton, 2010) might be facilitated by efficient and adequate resolution of complexity at multiple levels, contrasting the “rhomboidal modeling” approach (de Young et al., 2004) where functional complexity is only resolved at one particular trophic level of interest (Figure 1). In the following, we summarize indications for a fractal-like organization in the pelagic microbial food web and put forward the fractal hypothesis using conceptual infection and predation matrices to illustrate our idea.

One of the most conspicuous and well-documented characteristic of the pelagic food web, which might be linked to a fractal like organization, are normalized pelagic biomass-size spectra (Platt and Denman, 1977; Rodrigez, 1994; Marquet et al., 2005). Normalized biomass-size spectra can be summarized in a power law with a slope of −1, meaning that roughly equal biomass is present in each logarithmic size class (Sheldon et al., 1972). These findings became later known as the “linear-biomass hypothesis” (Marquet et al., 2005). Whereas the original formulations by Sheldon et al. (1972) were based on sparse data, the linear-biomass hypothesis has in recent years been supported by more data from plankton biomass-size spectra collected in different marine environments including estuaries, coastal seas and oligotrophic gyres (Rodrigez, 1994; Choi et al., 1999; Quinones et al., 2003; Irigoien et al., 2004; Tao et al., 2008; Ward et al., 2012). Data in these studies confirm a linear decrease in biomass across size classes with a slope of –1 when plotting the normalized biomass (Platt and Denman, 1977) against cell size (or alternatively abundance against cell volume). Although power laws do not necessarily imply underlying fractal structures, they are characteristic for fractals (Brown et al., 2002) and supportive of the notion of scale-invariance in the pelagic ecosystem. This would suggest that logarithmically spaced organism size classes may have comparable roles within their respective trophic levels.

Earlier attempts to explain the power law in the biomass-size spectra of pelagic food webs include the hypothesis that the roughly 10% efficiency of energy transfer between trophic levels, together with a reduction of metabolic rates by roughly 10% from one trophic level to the next, lead to the equal biomass per logarithmic size classes (Sheldon et al., 1972; Platt and Denman, 1978). However, these arguments are based on a linear food chain (Choi et al., 1999), which does not apply to the pelagic food web and the microbial loop in particular, which are highly interconnected networks. Based on allometric scaling laws and abundance distributions in different size classes, as described in the metabolic theory of ecology (Brown et al., 2004), Marquet et al. (2005) summarize how energy use in different size classes should be roughly equal and independent of body mass, leading to roughly equal biomass per size class. Although successfully unifying a proposed invariance in energy use within and biomass invariance across trophic levels in the pelagic ecosystem, this theory lacks the power to explain how several different types of equally sized organisms can coexist, i.e., how biodiversity can be maintained within a particular trophic level. We suggest that the Killing-the-Winner (KtW) mechanisms (Thingstad, 2000) could underlie a fractal-like organization of the pelagic food web and hence be responsible for the proposed scale-invariance.

**Figure 2:**
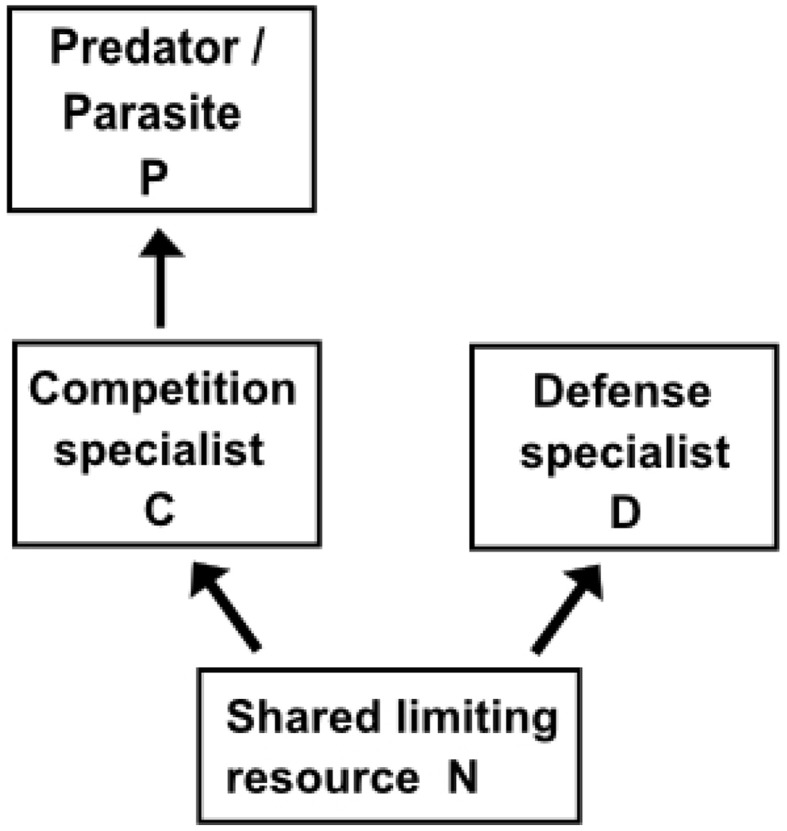
**Basic structure explaining the Killing-the-Winner (KtW) mechanism**. Coexistence of a superior competitor (competition specialist, C) and inferior competitor (defense strategist, D) based on a single limiting resource (N) is made possible due to selective top-down control of the competition strategist by either a predator or parasite (P).

**Figure 3:**
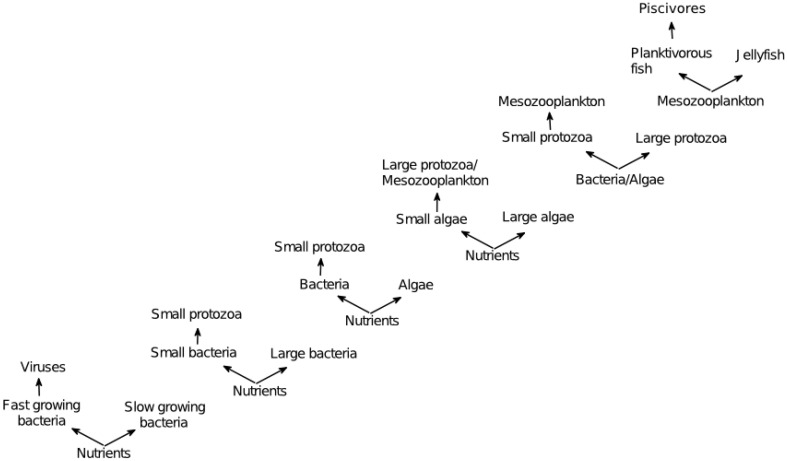
**Examples of KtW acting at different trophic levels; bacteria-virus communities (Bohannan and Lenski, 2000), protozoa-bacteria food webs (Matz and Jürgens, 2003), protozoa-bacteria-algae food webs (Pengerud et al., 1987), phytoplankton-metazoan food webs (Steiner, 2003), and fish-jellyfish systems (Haraldsson et al., 2012)**. Adapted from Våge (2014).

KtW has been shown to act repeatedly at different trophic levels and levels of phylogenetic resolutions (Pengerud et al., 1987; Bohannan and Lenski, 2000; Matz and Jürgens, 2003; Steiner, 2003). In the KtW mechanisms, top-down control through predation or parasitism on competitively superior species enables excessive resources to become available for inferior competitors. Given that the the system's total nutrient content is large enough, the mechanism hence allows coexistence of a superior and inferior competitor based on a single limiting resource (Figure 2). This principle is related to keystone predation described in macro-ecology (Våge et al., 2013), although keystone predation is used in more loose terms (Mills et al., 1993) than KtW, which is rooted in steady state analyses. Experimental verifications of the KtW mechanism have been made within bacteria-phage communities (Bohannan and Lenski, 2000), protozoa-bacteria food webs (Matz and Jürgens, 2003), protozoa-bacteria-algae food webs (Pengerud et al., 1987) and phytoplankton-metazoan food webs (Steiner, 2003). Together with the late application of the same principle to analyze jellyfish and zooplanktivorous fish competition in the Baltic Sea (Haraldsson et al., 2012), this provides evidence that KtW may be a generic mechanism controlling biodiversity through top-down control of strong competitors in similar manners on different levels of the pelagic food web (Figure 3).

**Figure 4:**
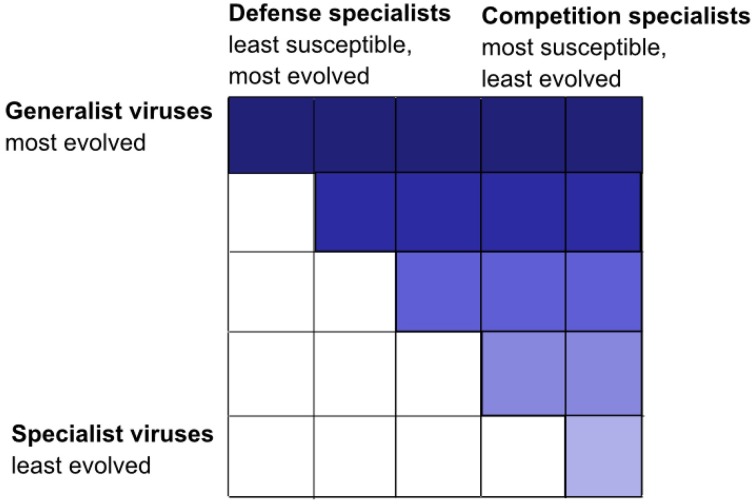
**Nested interaction matrix illustrated by the example of five different host and virus groups**. Positive infections are represented by a color-filled tile. Nested interactions results in a triangular matrix, in this case upper triangular with defense specialized hosts that are infected by few viruses only on the left and competition specialized hosts that are infected by most viruses on the right. Generalist viruses with a broad host range spectrum, able to infect all host groups, are on top (dark-colored interactions), and specialized viruses with a narrow host range able to infect few host groups only are at the bottom (light-colored interactions). Modified from Våge (2014). The same type of nested interaction matrix is conceivable from predator-prey interactions, where predators play the role analogous to viruses and prey to hosts.

The KtW mechanism is, according to the proposed hypothesis, responsible for an emergent, fractal like organization of trophic interactions in the pelagic microbial food web. For illustration, we first consider virus-host interactions, which represent “trophic interaction networks” at the highest level of phylogenetic resolution (i.e., on the levels of species and strains). Virus-host interactions tend to be highly specific (Lima-Mendez et al., 2015), but infection networks within a particular host community are characterized by varying infection and susceptibility ranges for viruses and hosts. Oftentimes, these infection networks can be summarized as nested infection patterns (Flores et al., 2011; Jover et al., 2013). In nested infection networks, generalist viruses represent the most evolved viral strains, infecting nearly all host strains within the host community, while specialist viruses represent more ancient strains able to infect ancient host strains only. Typically, ancient host strains are infected by most viruses including ancient types, whereas the more evolved host strains are defense specialists, susceptible to few, evolved viruses only. The resulting infection matrix is upper triangular (Figure 4). Nested infection can arrive through expanded host range coevolution, where hosts evolve to become resistant against existing viruses, while viruses evolve to infect the ever increasing number of newly evolved host strains (Lenski and Levin, 1985; Buckling and Rainey, 2002). The cost of being a generalist virus is assumed to be reduced virulence in terms of lower adsorption coefficients, while defensive, evolved host strains pay with lower competitive abilities (Flores et al., 2011).

**Figure 5:**
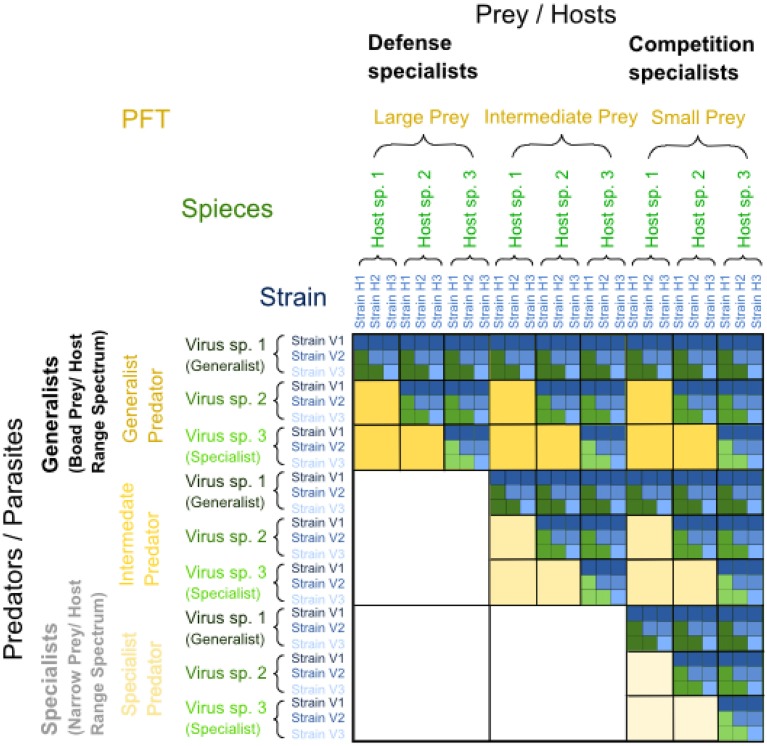
**Matrix illustrating examples of nested predator-prey and parasite-host interactions repeated at different trophic and phylogenetic levels of resolution, resulting in a fractal-like trophic interaction matrix**. Within each upper triangular matrix resulting from nested infection, defense specialized prey are found on the left and competition specialized prey on the right, while generalist predators or viruses with broad prey or host range spectra are found on top and specialized predators or viruses with narrow prey or host range spectra at the bottom. The yellow level represents a level of low phylogenetic resolution, such as plankton functional types (PFTs), where prey may be categorized into small, intermediate and large prey, where small prey are competition specialists and large prey are defense specialists. Predators on this yellow level of PFTs may be generalists eating prey of different sizes or specialists eating prey of a particular size only. The green level within the yellow level of PFTs represents an intermediate level of phylogenetic resolution, such as “species,” whereas the blue level within the green level of “species” represents a high level of phylogenetic resolution, such as “strains.” For a visual distinction of generalist vs. specialist strategies within each level of resolution, interactions with generalist predators or parasites are dark-colored, and those with specialists are light-colored. Modified from Våge (2014).

Host-range coevolution is intrinsically driven by the KtW mechanism, where new viruses evolve to control recently established host strains, which had gained improved defense against previously established viruses at the cost of reduced competitive ability (Thingstad et al., 2014). The result are arms-race dynamics, anticipated to occur simultaneously at other trophic levels and levels of phylogenetic resolution within the pelagic plankton food web. On higher trophic levels, we foresee the KtW mechanism to be expressed through predator-prey rather than virus-host interactions, with size-selective grazing (Cyr and Curtis, 1999; Hahn and Höfle, 1999; Thingstad et al., 2010) leading to nested predation networks on the level of plankton functional types. The result is a self-similar trophic interaction structure with subsets of upper triangular infection and predation matrices at different trophic levels and levels of phylogenetic resolution within the pelagic microbial food web (Figure 5).

Each inward level of the fractal-like representation of the trophic interactions in Figure 5 corresponds to a higher resolution of functional types. The yellow level has lowest resolution (e.g., plankton functional type, “PFT”). Focusing on the microbial part of the pelagic food web, we envision this level to be represented by microzooplankton grazers and their prey. Relatively large microbial predators such as heterotrophic dinoflagellates and ciliates may be classified as generalists, grazing on prey with a range of cell sizes including pico-, nano-, and microplankton (although for particular dinoflagellates species, narrower restrictions in terms of preferred cell size apply, Buskey, 1997), whereas the smaller heterotrophic nanoflagellates are more restricted to picoplankton prey and would classify as specialist predators. Among the prey, large potential prey such as diatoms and dinoflagellates are the most evolved forms and are typically protected against the most abundant microbial predators such as heterotrophic nanoflagellates and ciliates due to their big size (Thingstad et al., 2010). They generally give up on their competitive abilities relative to smaller prey (e.g., Tambi et al., 2009) and correspond to more evolved defense specialists in our example. Small prey with a longer evolutionary history, including bacteria and nanoflagellates, on the other hand, are under more severe grazing pressure by highly abundant microbial predators such as heterotrophic nanoflagellates and larger protozoa (Thingstad et al., 2010). Hence, they classify as more ancient competition specialists in our example.

Similarly, within the functional types described on the yellow “PFT” level, expanded host range coevolution is proposed to have led to nested infection and predation structures on the next level of higher phylogenetic resolution, e.g., “species” (green level in Figure 5). As an illustrative example, different prey species can be assumed to have evolved to emphasize defense during arms-race dynamics, while predator or parasite species may have co-evolved to become generalists with broader host ranges. An example would be grazing by flagellates causing a change in both morphology and taxonomy of the grazed bacterial community (Hahn and Höfle, 1999).

Within the green “species” level, nested infection networks may persist on the level of strains as described above for virus-host communities (blue level in Figure 5). Viruses are known to be most important for structuring the host community at this level of phylogenetic resolution due to their high specificity for host strains (Lima-Mendez et al., 2015) compared to predators. Strain-specific viruses with a narrow host range classify as specialized parasites, while other viruses with broader, species-specific host-ranges are generalist viruses (Flores et al., 2011; Jover et al., 2013). The notion of prokaryotic species and strains can be problematic (Doolittle and Zhaxybayeva, 2009); we here simply refer to these two terms as distinctive levels of phylogenetic relatedness with strains subdividing species.

Extending this concept beyond the purely microbial food web to include a next higher trophic level represented by mesozooplankton and their prey, copepods may be considered specialist predators compared to filter feeding appendicularia (chordata), which are less selective (Deibel, 1986) and thus would classify as generalist predators.

**Figure 6:**
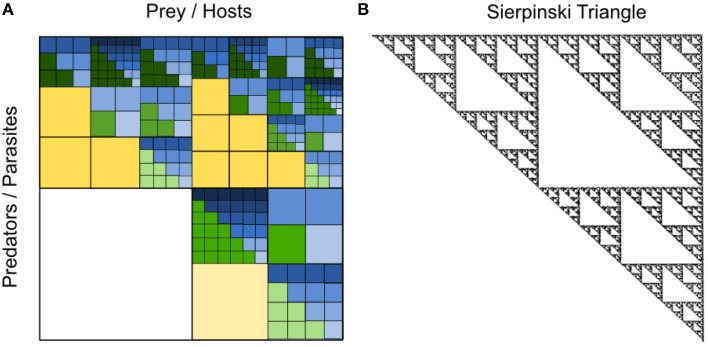
**(A)** Illustrative example of a nested predation and infection fractal with varying numbers of taxa at different levels of resolution. As in Figure 5, within each upper triangular matrix of the fractal, defense specialized prey are found on the left and competition specialized prey on the right, while generalist predators or viruses with broad prey or host range spectra are found on top and specialized predators or viruses with narrow prey or host range spectra at the bottom. For a visual distinction of interactions with generalist vs. specialist predators or parasites, interactions with generalists are dark-colored and those with specialists are light-colored within each level of resolution. The yellow level represents a level of low phylogenetic resolution, such as plankton functional types (PFTs), where prey may be categorized into small, intermediate and large prey, where small prey are competition specialists and large prey are defense specialists. Predators on this yellow level of PFTs may be generalists eating prey of different sizes or specialists eating prey of a particular size only. The green level represent an intermediate level of phylogenetic resolution, such as “species,” whereas the blue level represents a high level of phylogenetic resolution, such as “strains.” Adapted from Våge (2014). **(B)** Sierpinski triangle with a fractal structure similar to the hypothesized nested infection and predation network of the pelagic microbial food web. The Sierpinski triangle was generated by the chaos game as described in Barton (1990).

The examples chosen above illustrate the concept that we envision to underlie a fractal-like organization of the pelagic microbial food web. Clearly, natural food webs are far from being as regular as illustrated in Figure 5. The number of taxa between and within different trophic levels varies, and a fractal-matrix as outlined in Figure 6A may be more realistic. Interestingly, however, irrespective of the exact shape of the trophic interaction network, the above described mechanism leads to nested upper triangular interaction matrices with a conspicuous similarity to the well-known Sierpinski triangle (Figure 6B), as discussed below.

To summarize, we hypothesize that nested infection and predation networks in the pelagic microbial food web, repeated at different trophic levels and levels of phylogenetic resolution, may give a fractal-like organization of the food web, and that KtW is a central mechanism underlying the dynamics that lead to nested predator-prey and parasite-host networks.

## Discussion

Complexity and diversity shaped by evolution is one of the most distinct aspects and reasons for fascination in biology. Modern sequencing techniques give us insights into microbial ecosystems whose complexity has previously been obscure due to limited observations, revealing a biodiversity comprising most of the biosphere (Pace, 1997). While new molecular data on microbial diversity are being produced constantly, understanding microbial diversity remains a major challenge in ecology. In this article, we tried to address this challenge by putting the idea forward that much of biological complexity and diversity may be understood based on simple ecological concepts, using the KtW principle as an illustration of such a basic concept. Based on our considerations, we put forward a novel hypothesis that trophic interactions in the pelagic microbial food web may be organized in a fractal-like manner with structural resemblance to the Sierpinski triangle.

Scale-invariant features in ecosystems, such as population density scalings and biomass-size spectra (Allesina and Bodini, 2005; Marquet et al., 2005), upon which power-laws can be formulated, have in biology led to sporadic applications of fractal geometry, which was first popularized in the 1980s by Mandelbrot (1982). In marine ecology, scale-invariant biomass distributions in pelagic fish communities were for instance successfully described using a fractal biomass distribution model (Carcia-Gutierrez et al., 2009), but the study remained short of an explanation of biological mechanisms that could explain the proposed fractal structure. Although power-laws may be closely connected with fractals, understanding the mechanistic basis for power laws revealing scale-invariant features in ecosystems is obviously challenging, and it remains to be seen whether power laws merely summarize biological data or whether biology in fact is organized around underlying power-law functions (Vogel, 2003).

It is striking that the KtW mechanism has experimentally been observed at different trophic levels within the pelagic microbial food web, indicating self-similarity in food web organization. Nevertheless, the formulated hypothesis of a fractal-like organization of the pelagic food web through KtW mechanisms repeated at different trophic levels and levels of phylogenetic resolution clearly needs to be further scrutinized. Even-though biomass-size spectra of planktonic food webs (e.g., Choi et al., 1999; Quinones et al., 2003; Tao et al., 2008) confirmed Sheldons linear biomass hypothesis (Marquet et al., 2005) and are consistent with a scale-invariant structure of the pelagic food web, such power laws do not necessarily imply an underlying fractal structure (Brown et al., 2002). Also, while nested infection suggested here to generate a Sierpinski triangle-like organization of the pelagic microbial food web has been shown to prevail in many virus-host systems (Flores et al., 2011), more extensive, systematic and quantitative studies covering different trophic levels across the entire food web are required to build up large data sets for both predator-prey and parasite-host community interactions. Only in this way can we thoroughly challenge the fractal hypothesis of the pelagic microbial food web with the assumed nested nature of interactions across multiple scales of phylogenetic resolution and trophic interactions. In particular, cross-infection and prey-preference experiments for viruses and predators with varying host and prey-size ranges need to be set up. This requires both identification and culturing of suitable predator and prey communities and successful isolations of more viruses, including strain-specific viruses with narrow host ranges and viruses with broad host ranges infecting multiple strains and possibly different species.

To further corroborate the mechanisms behind the KtW theory, which underlies the hypothesized fractal-like organization of trophic interactions with nested interaction networks emerging across different scales, incubation experiments as conducted by Bouvier and del Giorgio (2007) should be repeated. By comparing bacterial diversity in natural communities before and after removing lytic viruses, Bouvier and del Giorgio detected a shift in dominance of host strains, where virus-suppressed “winners” of the competition for limiting nutrients become dominant in virus-free incubations. Another important way of testing the KtW theory will be to map growth rate spectra across different levels of phylogenetic resolutions. Using strain-specific virus-host interactions and a cost of resistance against viruses (Våge et al., 2013; Thingstad et al., 2014), recent versions of the KtW predict a dominance of slow growing defense specialists, in line with the conjecture that natural host communities are dominated by more resistant hosts, rather than by the strongest competitors (Suttle, 2007). Another testable prediction resulting from the KtW mechanism is a positive correlation between host growth rate spectra and viral abundances (Thingstad, 2000). Methods measuring activity on the single cell level such as the “click-chemistry” method for estimating single cell protein synthesis (Samo et al., 2014), in combination with quantitative flow cytometry data, will be important ways to test these predictions.

A major milestone toward a formalization of the fractal hypothesis would be to characterize the pelagic microbial food web by a fractal dimension, which would allow for simple descriptions of the pelagic ecosystem across a range of different scales using the identified fractal as a model for the food web structure (Halley et al., 2004). Depending on the number of distinguished functional types, species and strains, the fractal dimension of the upper triangular nested infection and predation matrices illustrated in Figures 5, 6A varies. For instance, the regular fractal in Figure 5 has a fractal dimension *D* of roughly 1.63 (calculated as *D* = log(6)/log(3), where 6 is the number of downscaled copies and the scaling factor is 1/3), closely resembling the Sierpinski triangle with a fractal dimension of roughly 1.58 [calculated as *D* = log(3)/log(2), see Box 1].

The slope of a fractal-based power-law can be understood as a measure of the underlying fractal's dimension (Carcia-Gutierrez et al., 2009). Identifying emergent power-law relationships based on the fractal infectivity and predation network hypothesized here would thus be crucial to the formulation of a fractal model of the pelagic microbial food web. We currently cannot see how the slope of –1 in the normalized biomass-size spectra discussed above might be linked to the fractal-dimension of the suggested infectivity and predation network. There might be other power laws instead that are associated with the speculated fractal-like organization of the infection and predation matrix, which we are currently unaware of. We anticipate that analyzing the number of interacting nodes on each level of resolution in an infection and predation matrix of an individual-based evolutionary model could possibly reveal power-law relationships between scale and occupancy (Hartley et al., 2003). The slopes of these power laws may subsequently be related to the fractal dimension of the hypothesized underlying nested infection and predation matrix.

Although structural departures from mathematical fractals is intrinsic to all natural objects and phenomena in various degrees, fractals are arguably the simplest method available to describe natural objects and patterns across several scales, and are hence useful as a null-hypothesis to identify and quantify scaling properties in nature (Halley et al., 2004). With our novel hypothesis that infection and predation networks in the pelagic microbial community have a fractal-like structure resembling the Sierpinski triangle, we hope to trigger further research into predator-prey and parasite-host interactions using cross-infection and prey-preference assays as discussed above, which will help testing this hypothesis. Besides the potential of facilitating end-to-end ecosystem models with a fractal-understanding of the pelagic food web, chaotic behavior in ecosystem dynamics with high sensitivity to initial conditions (Beninca et al., 2008) could then be better understood by seeing pelagic ecosystem complexity as a result of simple underlying mechanisms. Last but not least, regardless of whether the fractal hypothesis can be formalized or not, we hope that the consideration of how simple mechanisms could potentially explain much of the microbial diversity revealed through “omics” techniques can extend a (micro-)biologists mindset to approach the challenge of understanding microbial ecosystems, which in many ways can be seen as a model system for ecological theory in general (Jessup et al., 2004).

### Parallels between microbial and macro-ecology

Identifying processes found in different disciplines is an important step toward formulating unifying theories. As KtW and keystone predation are analogous concepts traditionally used to explain coexistence in microbial and macro-ecology, respectively (Våge et al., 2014), this mechanism is a unifying principle linking microbial and macro-ecology. Also, arms-race dynamics with underlying KtW mechanisms leading to increasing plankton size during the evolution of the marine microbial food web (Thingstad et al., 2010) can be understood on similar terms as what is known under “Cope's rule” in macro-ecology (Alroy, 1998; Hone and Benton, 2005); within the pelagic ecosystem, evolution of the planktonic food web is foreseen as a series of advancements in arms technology against predation during the last 3.5 billion years (Thingstad et al., 2010). Starting with heterotrophic prokaryotes, the addition of increasingly more complex organisms such as eukaryotic phytoplankton, ciliates, diatoms and copepods may be explained by antagonistic evolution where newly established predator-prey interactions drive evolution toward larger but competitively inferior prey (Thingstad et al., 2010, Figure 7). Within the pelagic microbial food web, increased body size has a high cost in terms of nutrient limitation and sinking rates as these are roughly proportional to the square of the cell radius (Tambi et al., 2009). Large organisms therefore generally loose in the competition for nutrients in diffusion limited environments and sink faster from productive surface waters. The establishment of large plankton groups must thus have been based on some novel advantage, most likely linked to selective advantage of large prey due to improved predation avoidance. Newly established prey with increased body size escaped the predation pressure of previously established predators adapted to predate on previously established, smaller prey. Increasing size of organisms over time in evolving lineages in macro-ecology is explained analogously in “Cope's rule” (Stanley, 1973; Alroy, 1998; Kingsolver and Pfennig, 2004). According to “Cope's rule,” such directional evolution can only persist when biotic factors are main drivers of selection in a relatively stable environmental setting, whereas extinction events driven by abiotic factors will disrupt this tendency (Stanley, 1973). Size-selective grazing over long periods of time in the relatively stable pelagic environment (compared to other habitats on Earth) may explain why size-structure in the planktonic food web is so clearly expressed.

**Figure 7:**
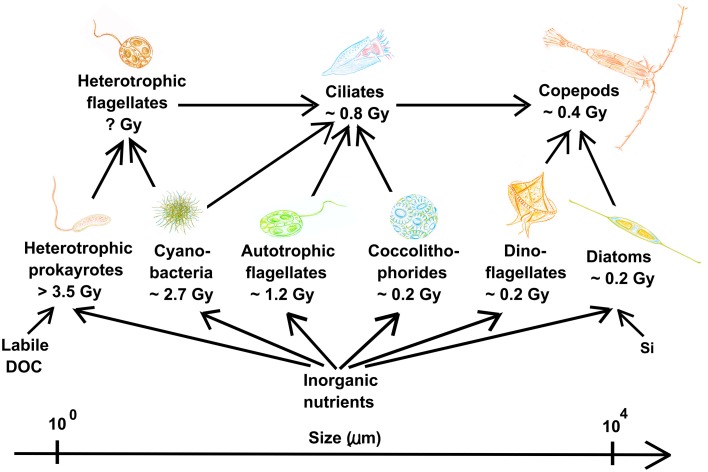
**Pelagic planktonic food web organized according to size and evolutionary appearance of plankton functional types**. Gy = 1 billion years before present. Viruses are not shown for simplicity but add an additional level of complexity to the trophic network within each plankton functional type, as described in the text. Note the organism size-span in this system covering several orders of magnitude. Modified from Thingstad et al. (2010).

Related to this discussion may be the huge diversification of metazoan body plans during the Cambrian explosion some 520 million years ago (Valentine et al., 1999). Although reasons for the relatively sudden diversification are still subject of debate (Marshall, 2006), it has been argued that explanations based on ecological interactions, in particular diversification through predation and arms race dynamics and niche saturation, have the potential to explain much of the diversification during the Cambrian explosion (Marshall, 2006). An increased number of needs an organism has to meet as a consequence of predator-prey interactions, such as successful predator avoidance, leads to a roughening of the fitness landscape with an increased number of local maxima and thus increased potential biodiversity (Niklas, 1994). A parallel between KtW as an important predator-prey and parasite-host interaction supporting diversity in the microbial food web and macro-ecological processes during the Cambrian explosion may thus exist.

### Scope of fractal hypothesis

The fractal-hypothesis of the pelagic microbial ecosystem presents a new perspective on the intriguing diversity and complexity found within the pelagic microbial community. It is reductionistic and by no means accountable for the full complexity of any biological system, yet it successfully addresses hierarchic aspects of complexity and diversity found within the microbial food web. Biodiversity within the same size-class of organisms warrants an explanation beyond size-based trophic interactions and allometric scalings, which form the basis of the “metabolic theory of ecology” (Brown et al., 2004). By describing how diversity may arise within any superimposed level of organization, our theory has in principle the power of explaining complexity to any desired level. It explains “within-community” diversity, both in terms of levels of phylogenetic resolution (i.e., high species diversity within PFTs and high strain diversity with species), and in terms of different trophic levels (i.e., diversity and organization on the level of PFTs). This is in contrast to other theories in ecology developed to explain community scale patterns based on underlying ecological and physiological constrains, such as the metabolic theory of ecology (Brown et al., 2004) that successfully addresses uniform biomass-size spectra in pelagic ecosystems (Marquet et al., 2005), but which falls short on addressing “within-community” diversity.

Lacking any description of the physical environment, our theory alone is not suitable for numerical simulations and predictions of ecosystem dynamics. Instead, the usefulness of the fractal-hypothesis lies in its reflection of a testable mechanistic theory, which is hypothesized to build a foundation for the multi-scale trophic and phylogenetic complexity in the pelagic microbial community.

## Conclusions

The perspective and hypothesis presented in this article is meant to illuminate how disentangling complexity in the pelagic microbial food web by extracting underlying mechanisms may help better understand microbial diversity. The KtW mechanism has been shown to play central roles in structuring pelagic food webs at different trophic levels and is here suggested to underlie infection and predation networks with fractal-like structures resembling the Sierpinski triangle. Although nature does not contain any mathematically precise fractals, fractals can be useful as a null-hypothesis to identify and quantify scaling properties in nature (Halley et al., 2004). As observations of fractal relationships in natural systems spanning scales of more than three orders of magnitude are very rare (Halley et al., 2004), the pelagic microbial ecosystem spanning at least four orders of magnitude in size (Finkel et al., 2010) is promising for further development and testing of the fractal hypothesis.

Achieving a formal understanding of the structure and mechanisms leading to a fractal-like organization of the pelagic ecosystem would have the potential of allowing modeling of ecosystem dynamics with adequate resolution at multiple scales. We speculate that analyzing the number of interacting nodes on each level of resolution in infection and predation matrices of individual-based evolutionary models might reveal power-law relationships between scale and occupancy, whose slope could be related to the fractal dimension of the underlying infection and predation matrix. This could then be used to model food webs using fractal-methods. Furthermore, although chaotic behavior in complex systems makes long-term predictions nearly impossible due to high sensitivity to initial conditions, understanding the fractal-like nature of the pelagic food web may help to understand ecosystem dynamics occurring at different scales.

Joint efforts of ecologists and mathematicians are needed to challenge the fractal-hypothesis through laboratory, field and theoretical studies, providing data and simulations on infection and predation networks. This will be required to formally characterize the nature of the potential fractal organization of the pelagic food web. If further scrutinizing should prove that a formalization of the fractal hypothesis is impossible in the near future, we advocate nevertheless a merit in having tried to approach a long-standing challenge of understanding microbial ecosystem complexity through innovative ways.

## Author contributions

TT conceived the original idea presented in this article, SV and TT developed the idea and theory further, SV wrote the manuscript and SV and TT revised the manuscript.

## Funding

The work was funded by the MINOS project financed by EU-ERC (proj.nr. 250254) and the University of Bergen, Norway.

### Conflict of interest statement

The authors declare that the research was conducted in the absence of any commercial or financial relationships that could be construed as a potential conflict of interest.
